# Supplementary material for: Physiological Health and Physical Performance in Multiple Chemical Sensitivity—Described in the General Population
Source: Int J Environ Res Public Health. 2022 Jul 25;19(15):9039. doi: 10.3390/ijerph19159039 (PMC9331319; doi:10.3390/ijerph19159039)
Supplement: Supplementary file 1 [file ijerph-19-09039-s001.zip › ijerph-1829648-supplementary.pdf]

**Table 1.** Participant characteristics and distributions (mean (SD) is presented unless otherwise stated).

| | MCS *All* (n=188) | $p^a$ | MCS with no comorbid FSD (N=109) | $p^a$ | Controls (n=7791) |
|---|---|---|---|---|---|
| Age | 53.6 (13.5) | 0.21 | 54.8 (13.6) | 0.04 | 52.7 (13.1) |
| Sex (% women) | 67 | <0.001 | 61 | 0.03 | 51 |
| ***Anthropometry*** | | | | | |
| Waist circumference (cm) | 91.6 (16.0) | 0.007 | 90.3 (14.3) | 0.32 | 88.9 (13.3) |
| Men >102 cm (%)[b] | 42 | 0.002 | 36 | 0.10 | 25 |
| Women >88 cm (%)[b] | 40 | 0.009 | 33 | 0.48 | 29 |
| Fat percentage | 33.2 (9.0) | <0.001 | 31.7 (8.7) | 0.003 | 29.1 (8.9) |
| BMI (kg/m2) | 27.3 (5.7) | <0.001 | 26.3 (4.9) | 0.35 | 25.9 (4.4) |
| % Normal weight | 40.9 | <0.001 | 46.8 | 0.14 | 47.0 |
| % Overweight | 30.8 | | 29.3 | | 37.2 |
| % Obese (class I-III) | 28.2 | | 23.8 | | 15.7 |
| ***Cardiorespiratory fitness*** | | | | | |
| Forced Vital Capacity (L), median (IQR) | 3.55 (1.08) | <0.001[Ŧ] | 3.60 (1.19) | <0.001[Ŧ] | 3.98 (1.43) |
| Forced Expiratory Volume s1 (L), median (IQR) | 2.73 (0.79) | <0.001 | 2.74 (0.74) | <0.001[Ŧ] | 3.07 (1.14) |
| FVC (L)/FEV1 (L) | 0.76 (0.09) | 0.06 | 0.76 (0.08) | 0.30 | 0.76 (0.10) |
| Systolic blood pressure (mmHg) | 127.2 (17.9) | 0.05 | 127.5 (17.5) | 0.17 | 129.8 (18.2) |
| Diastolic blood pressure (mmHg) | 78.4 (10.0) | 0.55 | 78.7 (10.2) | 0.90 | 78.8 (10.3) |
| ***Physical performance*** | | | | | |
| Hand grip test (kg), median (IQR) | 63.0 (31.0) | <0.001[Ŧ] | 67.2 (31.7) | <0.001[Ŧ] | 79.1 (24.7) |
| Step test (METS count) | 8.2 (2.3) | <0.001 | 8.5 (2.3) | <0.001 | 9.7 (2.6) |
| Self-perceived fitness (%good/very good) | 27 | <0.001 | 37 | 0.20 | 44 |
| ***Mental health factors*** | | | | | |
| Cohens Stress scale, median (IQR) | 13.0 (9.0) | <0.001[Ŧ] | 12.0 (8.5) | 0.001[Ŧ] | 9.0 (8.0) |
| Self-perceived health (% good/very good) | 27 | <0.001 | 38 | <0.001 | 56 |
| SCL Anxiety, median (IQR) | 4.0 (6.0) | <0.001[Ŧ] | 2.0 (4.0) | <0.001[Ŧ] | 1.0 (2.9) |
| SCL Depression, median (IQR) | 6.0 (10.5) | <0.001[Ŧ] | 4.0 (7.0) | <0.001[Ŧ] | 2.0 (4.9) |
| ***Other lifestyle factors*** | | | | | |
| Wake up early (% yes, often) | 69 | <0.001 | 43 | 0.01 | 33 |
| Cannot sleep (% yes, often) | 57 | <0.001 | 18 | 0.21 | 14 |
| Alcohol intake | | | | | |
| ≤ recommended for women, %[c] | 89 | 0.64 | 83 | 0.008 | 92 |
| ≤ recommended for men, %[c] | 85 | 0.17 | 82 | 0.42 | 87 |
| Smoking (yes, daily, or frequently) | 17 | 0.65 | 17 | 0.55 | 15 |
| Self-reported OPD diagnosed by MD, % | 9 | <0.001 | 8 | <0.001 | 2 |
| Self-reported asthma diagnosed by MD, % | 26 | <0.001 | 18 | 0.001 | 9 |

a Tested mean/median differences between MCS case status and controls using Kruskal-Wallis test for skewed variables[Ŧ] otherwise T-test. Chi Squared was applied for categorical variables. b Adapted based on recommended cutoff from Ref. [40]. 2021. American Heart Association. c Adapted based on recommended cutoff from Ref. [37]. 2010. The Danish National Board of Health. [40]. FSD Functional Somatic Disorder, FVC Forced Vital Capacity, FEV1 Forced Expiratory Volume first second, IQR Inter quartile range, OPD Obstructive pulmonary disease MD Medical doctor

**Table 2**. Association between anthropometry measures and MCS case status compared to controls.

| | β coefficient (95% CI) | | |
|---|---|---|---|
| | **Model 1** | **Model 2** | **Model 3** |
| **Controls** | 1.00 | 1.00 | 1.00 |
| **Waist circumference** (cm) | | | |
| *MCS All* | **4.28 (2.65, 5.90)** | **4.23 (2.52, 5.94)** | **3.81 (2.09, 5.54)** |
| *MCS with no comorbid FSD* | 2.05 (-0.06, 4.16) | **2.26 (0.07, 4.45)** | 2.07 (-0.11, 4.26) |
| **Body fat** (%) | | | |
| *MCS All* | **2.13 (1.18, 3.09)** | **2.25 (1.24, 3.26)** | **2.02 (1.00, 3.04)** |
| *MCS with no comorbid FSD* | 1.02 (-0.21, 2.27) | 1.18 (-0.10, 2.48) | 1.08 (-0.20, 2.38) |
| **BMI** ($kg/m^2$) | | | |
| *MCS All* | **1.53 (0.90, 2.16)** | **1.50 (0.85, 2.17)** | **1.36 (0.70, 2.03)** |
| *MCS with no comorbid FSD* | 0.49 (-0.33, 1.30) | 0.56 (-0.28, 1.40) | 0.49 (-0.35, 1.34) |

FSD Functional Somatic Disorder

Model 1: adjusted for sex, age, and age^2. Model 2: additionally, adjusted for alcohol, smoking, and sleep disturbances. Model 3: additionally, adjusted for Cohens perceived stress scale, depression, and anxiety.

**Table 3.** Association between cardiorespiratory fitness, physical performance and MCS *All* or MCS with no comorbid FSD compared to controls.

| | **β coefficient (95% CI)** | | |
|---|---|---|---|
| | **Model 1** | **Model 2** | **Model 3** |
| *Cardiorespiratory fitness* | | | |
| **FVC***(L) | | | |
| *MCS All* | **-0.21 (-0.29, -0.13)** | **-0.20 (-0.29, -0.12)** | **-0.20 (-0.27, -0.11)** |
| *MCS with no comorbid FSD* | **-0.12 (-0.23, -0.02)** | -0.09 (-0.20, 0.02) | -0.08 (-0.19, 0.02) |
| **FEV1***(L) | | | |
| *MCS All* | **-0.29 (-0.27, -0.12)** | **-0.18 (-0.26, -0.09)** | **-0.17 (-0.25, -0.09)** |
| *MCS with no comorbid FSD* | **-0.11 (-0.21,-0.02)** | -0.08 (-0.18, 0.02) | -0.07 (-0.17, 0.03) |
| **FEV1/FVC** | | | |
| *MCS All* | -0.01 (-0.02, 0.00) | -0.01 (-0.03, 0.00) | -0.01 (-0.03, 0.00) |
| *MCS with no comorbid FSD* | -0.008 (-0.02, 0.01) | -0.006 (-0.02, 0.01) | -0.006 (-0.03, 0.01) |
| **Systolic BP** (mmhg) | | | |
| *MCS All* | **-2.54 (-4.83, -0.25)** | -2.40 (-4.83, 0.03) | **-2.66 (-5.11, -0.21)** |
| *MCS with no comorbid FSD* | **-3.48 (-6.46, -0.50)** | **-3.86 (-6.97, -0.75)** | **-3.93 (-7.03, -0.82)** |
| **Diastolic BP** (mmhg) | | | |
| *MCS All* | 0.12 (-1.25, 1.51) | 0.34 (-1.12, 1.82) | 0.19 (-1.29, 1.68) |
| *MCS with no comorbid FSD* | 0.05 (-1.74, 1.85) | -0.02 (-1.91, 1.85) | -0.08 (-1.97, 1.80) |
| *Physical performance* | | | |
| **Handgrip** (pounds) | | | |
| *MCS All* | **-5.64 (-7.70, -3.58)** | **-4.54 (-6.71, -2.36)** | **-3.98 (-6.18, -1.78)** |
| *MCS with no comorbid FSD* | -2.42 (-5.10, 0.25) | -1.88 (-4.68, 0.90) | -1.63 (-4.42, 1.16) |
| **Steptest** (Mets) | | | |
| *MCS All* | **-1.20 (-1.59, -0.82)** | **-1.17 (-1.57, -0.77)** | **-1.07 (-1.47, -0.67)** |
| *MCS with no comorbid FSD* | **-0.70 (-1.18, -0.22)** | **-0.73 (-1.22, -0.24)** | **-0.68 (-1.16, -0.19)** |

FSD Functional Somatic Disorder, FVC Forced Vital Capacity, FEV1 Forced Expiratory Volume first second, BP Blood pressure. Model 1: adjusted for sex, age, and age^2. Model 2: additionally, adjusted for alcohol, smoking, and sleep disturbance. Model 3: additionally, adjusted for Cohens perceived stress scale, depression, and anxiety. *FVC and FEV1 also adjusted for height in all models.

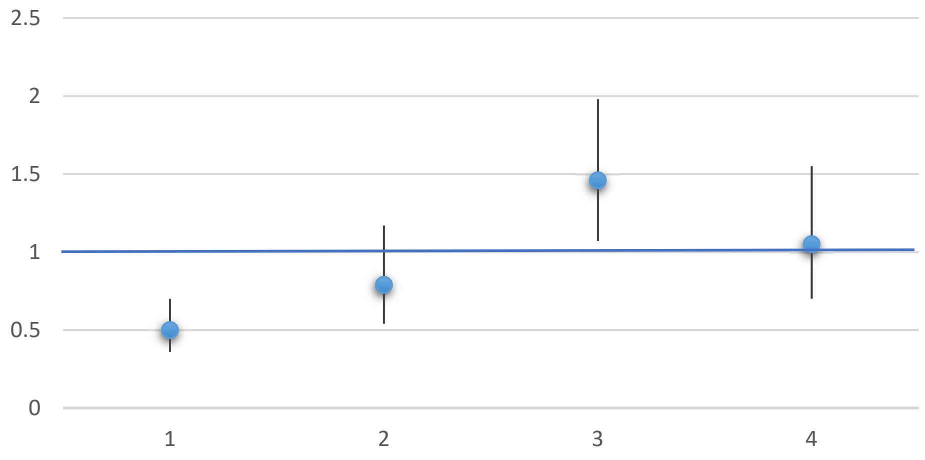


Figure 1. Odds ratio of high self-perceived fitness and overweight in MCS *All* or MCS with no comorbid FSD.

1: OR (95%CI) of **high self-perceived fitness** in MCS *All* adjusted for sex and age.
2: OR (95%CI) of **high self-perceived fitness** MCS with no comorbid FSD adjusted for sex and age.
3: OR (95%CI) of **BMI > 25 kg/m2** in MCS *All* Adjusted for sex, age, and age^2.
4: OR (95%CI) of **BMI > 25 kg/m2** in MCS with no comorbid FSD Adjusted for sex, age, and age^2.
FSD Functional Somatic Disorder.
High self-perceived fitness: participants who answered "good" or "very good" on a 5-point scale for self-perceived fitness.
